# A Smartphone-Based Acoustic Machine Learning Pipeline for Detecting Suicidal Ideation: Case-Control Model Development and Validation Study

**DOI:** 10.2196/92646

**Published:** 2026-09-15

**Authors:** Min Lyu, Lixin Tan, Jun Xiao, Heqing Huang, Fangjian Liu, Jiahui Qi, Tian Huang, Jinyu Lei, Zhihui Zhao, Tianxiang Jiang, Zhu Liu, Xueqian Wang, Jiang Zhong, Zhengzhi Feng

**Affiliations:** 1 Department of Medical Psychology, Army Medical University, No. 30 Gaotanyan Main Street, Shapingba District, Chongqing, 400038, China, +86-16623460852; 2 Mental Health Education and Counseling Center, Chongqing University, Chongqing, China; 3 Department of Cardiovascular Medicine, Chongqing Emergency Medical Center/The Fourth People's Hospital of Chongqing, Chongqing, China; 4 Department of Medical Psychology, First Affiliated Hospital of Army Medical University, Chongqing, China; 5 Faculty of Health and Wellness, City University of Macau, Macau, China; 6 College of Computer Science, Chongqing University, Chongqing, China; 7 College of Medicine, Chongqing University, Chongqing, China

**Keywords:** suicidal ideation, speech acoustics, machine learning, university students, cross-validation, artificial intelligence, AI

## Abstract

**Background:**

Suicidal ideation (SI) among university students is a growing public health concern. Self-report screening can be limited by concealment and delayed disclosure. We evaluated a leakage-resistant, proof-of-concept pipeline to detect SI from standardized smartphone-recorded speech.

**Objective:**

This study aimed to extract acoustic markers from brief smartphone-based reading tasks and develop machine learning models for suicide risk prediction in university students, enabling low-cost, scalable early screening to support campus mental health services.

**Methods:**

Questionnaire data and speech recordings were collected via a WeChat mini program. After screening and clinical confirmation, 96 participants (n=48, 50% with SI; n=48, 50% controls) were included. Age and sex were evaluated as potential confounders. Each participant read 16 standardized sentences. Acoustic features were extracted using openSMILE (version 3.0.2), yielding a 570D feature vector per utterance. To prevent leakage from multiple recordings per speaker, we used participant-level 5-fold cross-validation, assigning all recordings from each participant to a single fold. Seven machine learning algorithms were evaluated using area under the curve (AUC), accuracy, and *F*_1_-score.

**Results:**

Acoustic-based models discriminated participants with SI from control participants. The SI group was significantly older than the control group (*P*=.001). Random forest achieved an AUC of 0.813 (accuracy=0.748), and naive Bayes achieved an AUC of 0.806 (accuracy=0.757). Feature families related to pitch, mel-frequency cepstral coefficients, and harmonicity contributed to model performance.

**Conclusions:**

Standardized read speech captured via smartphones shows preliminary feasibility for SI discrimination under a leakage-aware evaluation design. External validation and testing with more naturalistic speech are warranted.

## Introduction

Suicide is recognized by the World Health Organization as a major public health concern, underscoring the importance of early detection and timely intervention [1]. Despite advances in mental health services, conventional suicide risk assessment—largely reliant on self-report scales and clinical interviews—has well-documented limitations, including susceptibility to social desirability and disclosure bias, limited sensitivity to rapid fluctuations in suicidal ideation (SI), and poor scalability in resource-constrained university settings [2,3]. These challenges motivate the development of objective, noninvasive indicators that could support continuous and large-scale screening.

University students, as emerging young adults, face academic demands, interpersonal adjustment, and career-related pressures that may heighten psychological distress and vulnerability to suicidal thoughts and behaviors [4]. Epidemiological studies report relatively high prevalence of SI in university samples [5-9]. Moreover, limited social support and restricted access to mental health services have been identified as important contributors to risk in this population [10-12]. Beyond prevalence estimates, recent work highlights the interplay of psychological, social, and cultural influences on suicidality, informing early identification and campus-based intervention strategies [13,14]. However, objective and efficient risk assessment approaches suitable for real-world university contexts remain necessary.

In acoustic analysis, there is accumulating evidence suggesting that speech may provide objective and scalable signals associated with suicidality and suicide risk. In prior studies, individuals at elevated suicide risk have been reported to exhibit atypical vocal patterns, including increased fundamental frequency (F0) variability, higher jitter and shimmer, altered mel-frequency cepstral coefficient (MFCC) profiles, and differences in spectral energy distribution [15,16]. These acoustic deviations are plausibly linked to underlying mechanisms implicated in suicidality, such as heightened emotional dysregulation, physiological stress responses, and increased cognitive load [17-19]. Consistent with this literature, a systematic review of acoustic machine learning studies identified jitter, F0, MFCCs, and power spectral density as among the most frequently used and potentially discriminative features while emphasizing substantial methodological heterogeneity (eg, recording tasks and feature extraction pipelines) that may limit generalizability [20].

With advances in AI and machine learning, an increasing number of studies have leveraged acoustic features (eg, MFCCs, formants, jitter, and energy) to predict suicide risk. Recent studies have reported promising results using diverse speech data and modeling approaches. Min et al [21] achieved 79% accuracy for within-person prediction of worsening suicidality and 69% accuracy for between-person classification. Su et al [22] achieved 75% accuracy using audio segments from crisis hotline calls. Krautz et al [23] analyzed the voice recordings of individuals who completed suicide and achieved 76% accuracy. Ding et al [24] constructed a crisis hotline suicide risk speech dataset and achieved 96% accuracy using a deep learning model. Lin et al [18] achieved 77.82% accuracy combining acoustic and word frequency features under negative emotional conditions. Zhu et al [25] achieved an area under the curve (AUC) of up to 1.00 for SI classification. Despite these encouraging results, evidence in university student populations remains limited, and variability in recording contexts and analytic pipelines continues to challenge robust and transferable models [20].

Therefore, we designed a study focusing on university students to extract key acoustic markers from brief smartphone-based reading tasks and develop suicide risk prediction models using machine learning algorithms. By using a low-cost and easily deployable recording protocol, this approach aims to support scalable early screening that could be integrated into campus mental health services to facilitate timely, data-informed prevention and intervention.

## Methods

We conducted a two-stage method development study to (1) develop and validate a set of standardized sentences for Chinese university students and (2) develop an acoustic-based machine learning pipeline for detecting SI. Importantly, the sentence validation cohort and the model development cohort were mutually exclusive to ensure independent evaluation across stages and prevent data leakage.

### Ethical Considerations

This study received approval from the ethics committee of the Fourth People’s Hospital of Chongqing (approval 2025; ethical review 73). Informed consent was obtained from all participants and/or their parents or legal guardians, in line with the Declaration of Helsinki. Participants with SI were further evaluated and intervened accordingly.

### Stage 1: Corpus Validation

A total of 54 university students aged 18 to 25 years were recruited via an online survey platform to rate candidate sentences. These participants did not take part in stage 2. A pool of 50 candidate sentences was created to cover three domains: (1) positive-valence statements, (2) negative-valence expressions, and (3) neutral factual statements. The positive-valence statements were imbued with Chinese traditional virtues and socialist core values to strengthen cultural resonance. To elicit diverse prosodic patterns, sentences were varied across syntactic moods (declarative, interrogative, and exclamatory). Sentence length was restricted to 100 Chinese characters or less, with a reading duration of less than 30 seconds to maintain feasibility and reduce fatigue.

Participants rated all 50 sentences on 9-point Likert scales for pleasure, arousal, and dominance, generating quantitative affective profiles. Two licensed clinical psychologists independently evaluated the sentences for readability, emotional expressiveness, and categorical balance. On the basis of consensus, 12 sentences (4 per valence domain) were selected. Four suicide-related statements adapted from validated instruments [26] were then added, resulting in a final set of 16 standardized prompts (Table S1 in Multimedia Appendix 1).

### Stage 2: Model Development

We conducted a case-control study among university students in southwest China using a sequential screening-and-confirmation process. Data were collected via a WeChat mini program, yielding 747 questionnaire responses and 771 speech recording submissions. Speech recordings were collected on participants’ personal smartphones in familiar, low-stress environments to enhance comfort and ecological validity [27]. Raw audio files were stored in encrypted, access-controlled servers and were used only for acoustic feature extraction [28]. After excluding incomplete entries and screening for audio quality, 715 participants remained eligible for SI screening. SI was initially screened using the Self-Rating Idea of Suicide Scale (SIOSS; threshold ≥12). To reduce misclassification due to concealment, individuals scoring 4 or more on the SIOSS concealment subscale were excluded. Participants who were screened as positive then underwent counselor-administered clinical confirmation using the Columbia-Suicide Severity Rating Scale, requiring an ideation subscale score of 2 or more. This process identified 48 clinically confirmed SI cases. From those who were screened as negative on the SIOSS (<12), we randomly selected 48 controls (1:1) using a computer-generated random sample. Age and sex were compared between groups and were adjusted for in subsequent analyses. The final analytic sample comprised 96 participants. Each participant recorded 16 standardized utterances in quiet settings, yielding 1536 recordings for model development.

### Audio Preprocessing and Reading Compliance Checking

All recordings underwent a standardized preprocessing pipeline. Audio was first enhanced using the Model Scope Acoustic Noise Suppression model. To verify reading compliance and exclude off-target recordings, speech was transcribed using the Google Speech-to-Text API (automatic language detection enabled). Transcripts were aligned with target sentences using a dual-weighted fuzzy matching procedure (FuzzyWuzzy library; 60% token set similarity and 40% edit distance similarity); a composite similarity score above 65% indicated a valid match. All matches were manually reviewed, and ambiguous cases were adjudicated by trained researchers. Transcripts were used solely for quality control and compliance checking; no linguistic features were included in model training. Recordings were then converted from MP3 (44.1 kHz; stereo) to WAV (16 kHz; mono; 16 bits) file formats using FFmpeg. After preprocessing, downstream analyses were conducted exclusively on deidentified acoustic feature values rather than raw speech signals.

### Acoustic Feature Extraction

Acoustic features were extracted using openSMILE[29] (version 3.0.2; audEERING GmbH [43] with the emobase and emobase2010 configurations, yielding a 570D feature vector for each utterance. The feature space comprised 30 low-level descriptors summarized by 19 functionals, covering intensity and loudness, spectral properties (eg, MFCCs), pitch-related measures (F0 and derived measures), voice quality indexes (jitter, shimmer, and harmonic-to-noise ratio [HNR]), and short-term signal characteristics (eg, zero-crossing rate). Full feature definitions and naming conventions are provided in Multimedia Appendix 1.

### Cross-Validation Design and Leakage-Aware Evaluation

All analyses were implemented in R (version 4.3.3; R Foundation for Statistical Computing). To prevent optimistic bias due to within-participant leakage (ie, multiple recordings per participant), we used participant-level stratified 5-fold cross-validation, assigning all 16 utterances from the same participant to a single fold [30,31]. Stratification was performed by case-control status and sex to preserve balance across folds. Age was evaluated as a potential confounder; given the narrow age range and the focus on acoustic-only feature sets, models were primarily trained on speech features.

Within each training fold, we applied the following preprocessing steps: (1) removal of constant features, (2) elimination of highly correlated feature pairs (|*r*|≥0.90), and (3) minimum-maximum normalization using parameters derived from the training data and applied unchanged to the corresponding test fold. All data-driven steps—including normalization, feature filtering, feature selection, and any hyperparameter tuning—were performed using training data only.

All performance metrics were computed at the participant level (N=96). Participant-level predictions were obtained by averaging the 16 utterance-level feature vectors recorded for each participant to form a single participant-level feature representation, which was then used as input to the model. Accordingly, each confusion matrix adds up to 96.

### Feature Selection

Feature selection was conducted within each training fold using a prespecified mixed-ranking strategy integrating 3 criteria: random forest (RF) variable importance (50%), feature-outcome correlation (30%), and ANOVA statistics (20%) [32,33]. Features were ranked by aggregated scores, and the top 16 features were retained for model training in each fold. The selected features predominantly captured prosodic, spectral, and voice quality characteristics (eg, MFCC, Line Spectrum Pairs (LSP), F0 and derived measures, and HNR).

### Model Validation

We evaluated 7 machine learning algorithms: RF, elastic net generalized linear model (GLMNET), Extreme Gradient Boosting (XGB), support vector machine (SVM; radial basis function kernel), k-nearest neighbor (KNN), naive Bayes (NB), and a feed-forward neural network (NNET).

Given the modest sample size (N=96), hyperparameter settings were intentionally conservative to reduce overfitting. Analyses were conducted using standard R packages: *randomForest* (RF), *glmnet* (GLMNET), *xgboost* (XGB), *e1071* (SVM), *caret* (KNN and NNET), and *naivebayes* (NB). Hyperparameter settings were as follows: RF used 300 trees with a minimum node size of 10; GLMNET used an α of 0.5 with λ selected via inner cross-validation; XGB used max_depth of 3, learning_rate of 0.1, and a subsample of 0.8 with L1 and L2 regularization (α=0.5; λ=1); SVM used a radial basis function kernel with C of 1; KNN and NNET were fit via *caret* with limited inner resampling for parameter selection; and NB used default parameters without tuning. All tuning and model fitting was confined to training folds.

### Performance Evaluation

Model discrimination was assessed using AUC as the primary metric alongside accuracy, sensitivity, specificity, precision, negative predictive value, and *F*_1_-score. Receiver operating characteristic curves and confusion matrices were used to summarize discriminative performance and error patterns. Metrics were defined as follows:


(1) $$\text{Accuracy=}\frac{\text{TP+TN}}{\text{TP+ TN + FP + FN}}$$

(2) $$\text{Sensitivity=}\frac{\text{TP}}{\text{TP+ FN}}$$

(3) $$\text{Specificity=}\frac{\text{TN}}{\text{TN}\text{+ FP}}$$

(4) $$\text{Precision=}\frac{\text{TP}}{\text{TP}\text{+ FP}}$$

(5) $$\text{NPV =}\frac{\text{TN}}{\text{TN }\text{+ FN}}$$

(6) $$F1=2\times \frac{\text{Precision}\times \text{Sensitivity}}{\text{Precision}+\text{Sensitivity}}$$

### Feature Stability

Feature stability was quantified by the frequency with which each feature was selected across cross-validation folds. Features selected in 80% or more of folds were classified as stable [34]. All preprocessing parameters were derived from training data and applied identically to test data to ensure reproducibility.

## Results

### Overview

The final sample comprised 96 participants (n=48, 50% per group) aged 16 to 25 years (mean age 18.76, SD 1.48 years), with 42.7% (n=41) female participants overall. Gender distributions were comparable between groups (χ²₁=0.17; *P*=.68). Age differed between groups, with the SI group being older than the control group (SI: mean 19.25, SD 1.78 years; controls: mean 18.27, SD 0.92 years; *t*_94_=−3.39; *P*=.001; Table 1).

**Table 1.** Demographics of the participants (N=96).

Variables	Overall	SI^a^ group (n=48)	Control group (n=48)	*P* value
Age (y), mean (SD)	18.76 (1.48)	19.25 (1.78)	18.27 (0.92)	.001
Sex, n (%)				.68
Male	55 (57.3)	29 (60.4)	26 (54.2)	
Female	41 (42.7)	19 (39.6)	22 (45.8)	

a SI: suicidal ideation.

### Model Performance

**Figure 1.**
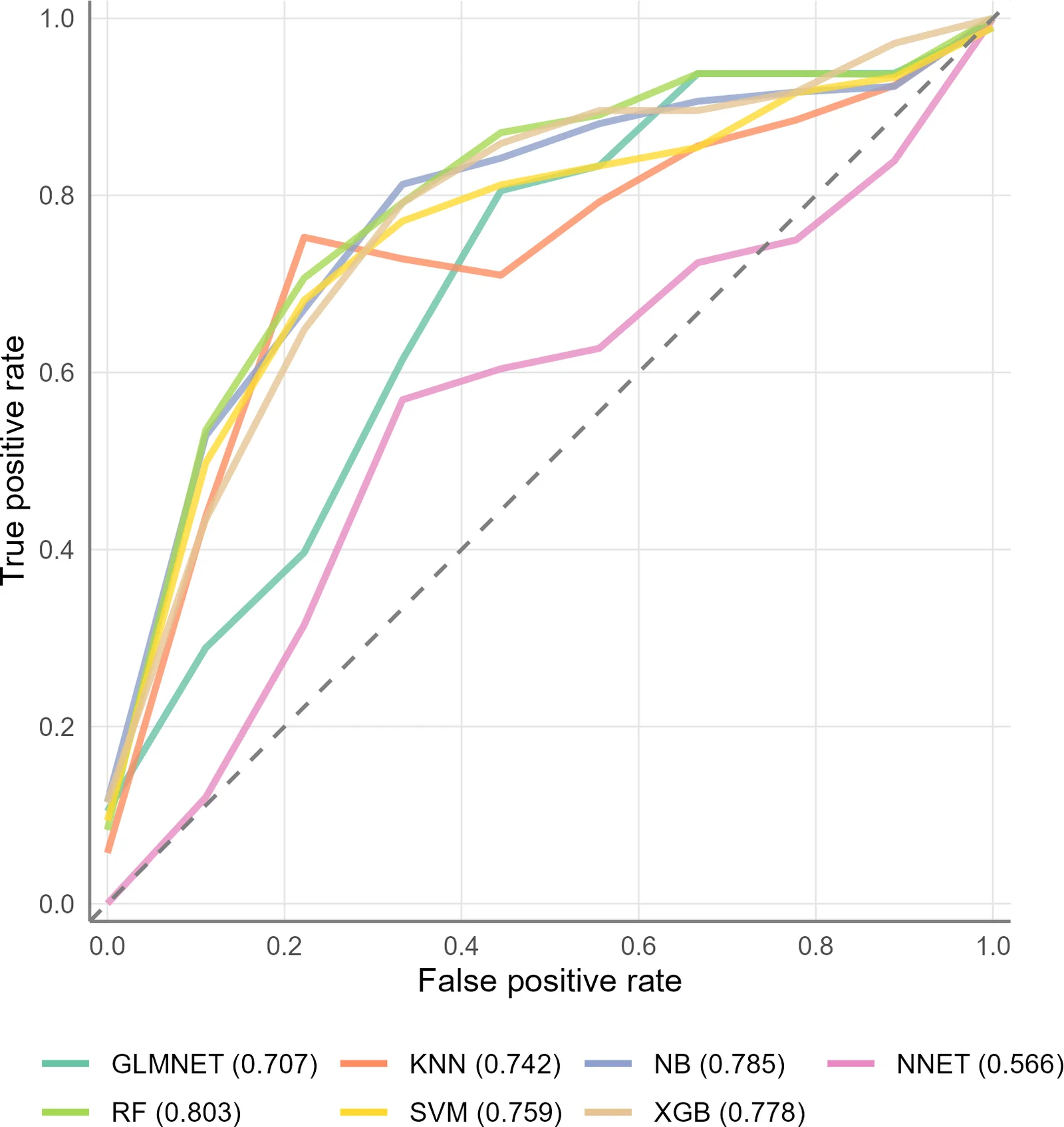
Receiver operating characteristic curves for the 7 machine learning models (random forest [RF], elastic net generalized linear model [GLMNET], Extreme Gradient Boosting [XGB], support vector machine [SVM], k-nearest neighbor [KNN], naive Bayes [NB], and feed-forward neural network [NNET]). RF achieved the highest area under the curve (AUC), whereas NNET showed the lowest AUC.

**Figure 2.**
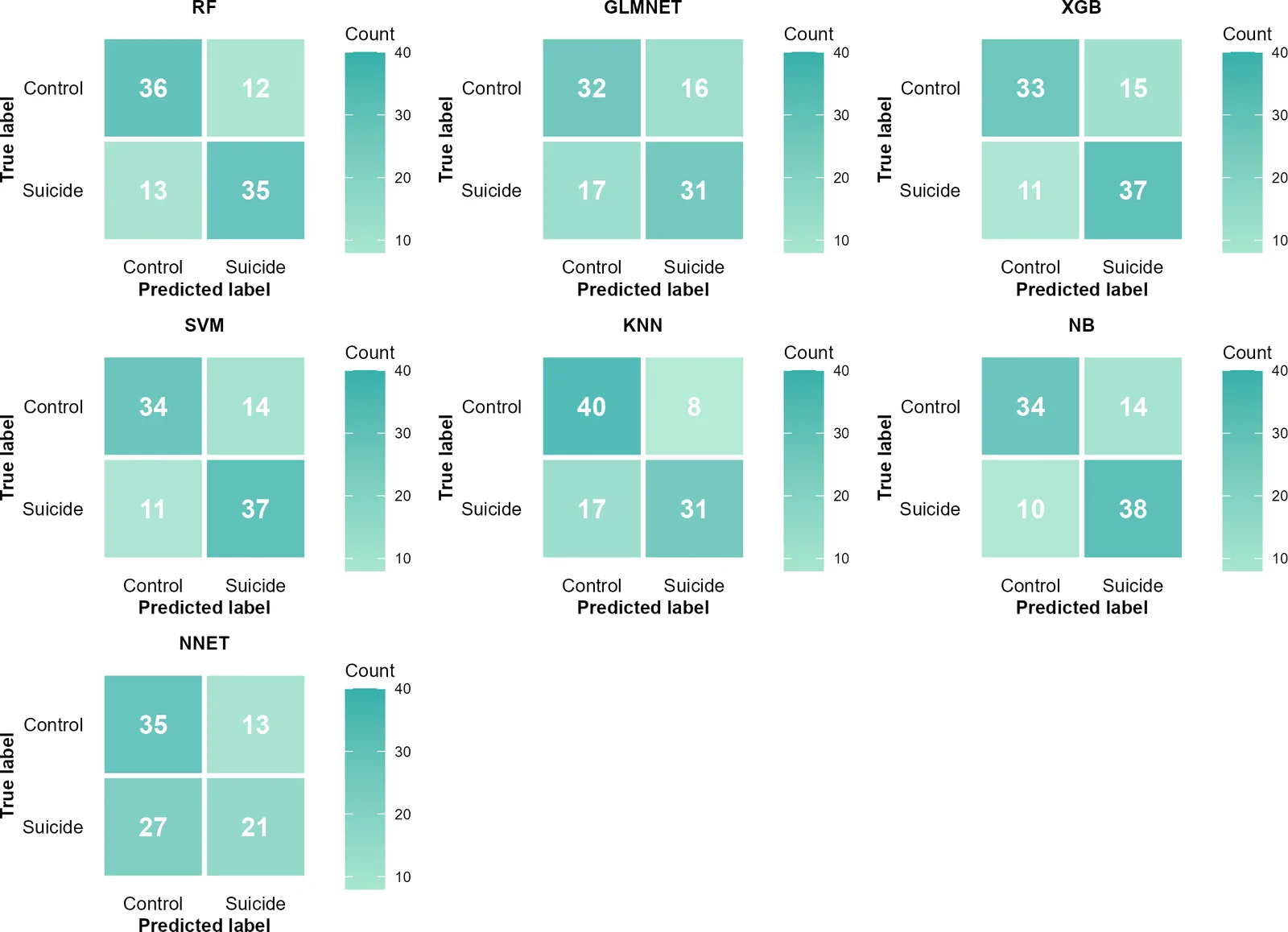
Confusion matrices for the 7 models (random forest [RF], elastic net generalized linear model [GLMNET], Extreme Gradient Boosting [XGB], support vector machine [SVM], k-nearest neighbor [KNN], naive Bayes [NB], and feed-forward neural network [NNET]) at the participant level (N=96). Rows indicate true labels (“control” and “suicide”), and columns indicate predicted labels (“control” and “suicide”); cell values are counts. Treating “suicide” as the positive class, the 4 cells correspond to true negatives (control correctly identified as control), false positives (control incorrectly identified as suicide), false negatives (suicide cases incorrectly identified as control), and true positives (suicide cases correctly identified as suicide).

As shown in Table 2, under participant-level, leakage-aware stratified 5-fold cross-validation, 6 of the 7 models achieved acceptable discrimination (AUCs>0.70), whereas NNET performed less well (AUC=0.629). RF yielded the best overall performance (AUC=0.813; accuracy=0.748; sensitivity=0.730; specificity=0.782). NB ranked second (AUC=0.806) and achieved the highest accuracy (0.757). XGB (AUC=0.781) and SVM (AUC=0.772) also showed good discrimination. KNN achieved the highest specificity (0.855) but lower sensitivity (0.645), whereas GLMNET showed more modest discrimination (AUC=0.729). Receiver operating characteristic curves and confusion matrices are shown in Figures 1 and 2, respectively.

**Table 2.** Predictive performance of the 7 machine learning models.

Models	AUC^a^ (95% CI)	Accuracy	Sensitivity	Specificity	Precision	NPV^b^	*F*_1_-score
RF^c^	0.813 (0.77-0.84)	0.748	0.730	0.782	0.772	0.725	0.747
GLMNET^d^	0.729 (0.64-0.81)	0.671	0.650	0.709	0.697	0.654	0.666
XGB^e^	0.781 (0.73-0.83)	0.738	0.770	0.727	0.740	0.752	0.746
SVM^f^	0.772 (0.71-0.83)	0.748	0.770	0.745	0.763	0.743	0.759
KNN^g^	0.757 (0.70-0.80)	0.740	0.645	0.855	0.811	0.691	0.715
NB^h^	0.806 (0.72-0.88)	0.757	0.790	0.745	0.769	0.761	0.770
NNET^i^	0.629 (0.56-0.72)	0.569	0.430	0.732	0.645	0.548	0.500

a AUC: area under the curve.

b NPV: negative predictive value.

c RF: random forest.

d GLMNET: elastic net generalized linear model.

e XGB: Extreme Gradient Boosting.

f SVM: support vector machine.

g KNN: k-nearest neighbor.

h NB: naive Bayes.

i NNET: feed-forward neural network.

Beyond aggregate metrics, an analysis of the confusion matrices (Figure 2) revealed specific patterns of misclassification. For the best-performing model (RF), while it correctly identified 72.9% (35/48) of the SI cases, 27.1% (13/48) of the participants with SI were incorrectly classified as controls (false negatives). Conversely, 20.8% (10/48) of the controls were misclassified as SI (false positives). These results indicate that, while the model captures core acoustic markers of distress, 27.1% (13/48) of SI cases remained undetected, highlighting the current limitations of a purely acoustic approach in this cohort.

### Feature Stability

The 20 most frequently selected features across cross-validation folds are shown in Figure S1 in Multimedia Appendix 1. Eight features were selected in at least 4 folds, indicating relatively consistent selection across resamples. These features predominantly reflected pitch variability (F0-related indexes), spectral shape (MFCC kurtosis), and harmonicity (HNR; Tables S2-S4 in Multimedia Appendix 1).

### Univariate Associations With SI Status

To characterize group differences for the robust feature set, we conducted independent-sample 2-tailed *t* tests and correlation analyses with false discovery rate (FDR) correction (Tables S2-S4 in Multimedia Appendix 1). After FDR correction, 7 of the 8 robust features remained significantly associated with SI status. Compared with the control group, the SI group showed higher values in 6 features—F0_sma_iqr2-3, F0env_sma_stddev, F0env_sma_iqr1-2, F0env_sma_linregerrQ, mfcc_sma [5]_kurtosis, and mfcc_sma [12]_kurtosis—whereas HNR_sma_iqr1-2 was lower in the SI group. Effect sizes were in the medium to large range (Cohen *d*=0.44‐0.85). Correlation analyses showed a consistent pattern: the 6 F0 variability and MFCC kurtosis features correlated positively with SI status (*r*=0.28‐0.40), whereas HNR_sma_iqr1-2 was negatively correlated (*r*=−0.22). HNR_sma_stddev did not survive FDR correction.

### Multivariable and Sentence Type Effects

Logistic regression analyses corroborated the univariate findings. Higher values of F0 variability indexes and MFCC kurtosis were associated with increased odds of SI group membership, whereas HNR_sma_iqr1-2 was associated with decreased odds. To facilitate interpretability, standardized odds ratios (ORs; per 1 SD increase) were reported: MFCC kurtosis features showed standardized ORs of 1.94 and 2.25, standardized ORs for F0-related features ranged from 2.03 to 2.50, and HNR_sma_iqr1-2 showed a standardized OR of 0.62 (Tables S2-S4 in Multimedia Appendix 1).

Mixed-effects models further indicated that sentence type accounted for substantial variance in most acoustic features (Figure S2 in Multimedia Appendix 1). Significant group effects were observed for mfcc_sma [5]_kurtosis (*F*_1,94_=9.048; *P*=.003) and mfcc_sma [12]_kurtosis (*F*_1,94_=7.718; *P*=.007), with nonsignificant sentence type effects and interactions (*P*>.05 in all cases). F0-related features showed both group effects (*F*_1,94_=10.296‐17.373; *P*<.001 to *P*=.002) and sentence type effects (*F*_3,1434_=13.256‐75.388; *P*<.001) without evidence of group × sentence type interactions. For HNR features, sentence type effects were pronounced (*F*_3,1434_=5.684 and 216.477; *P*<.001), whereas group effects were weak or marginal (HNR_sma_stddev: *F*_1,94_=2.593 and *P*=.11; HNR_sma_iqr1-2: *F*_1,94_=4.664 and *P*=.03), and interactions were nonsignificant.

### Model Interpretability

To interpret model behavior, we examined RF feature importance and visualized marginal effects using partial dependence plots. F0 variability features ranked among the most influential predictors (Figure S3 in Multimedia Appendix 1). Partial dependence plots suggested nonlinear relationships between feature values and predicted SI risk for several features (Figure S4 in Multimedia Appendix 1), whereas HNR_sma_iqr1-2 showed a nonmonotonic pattern.

**Figure 3.**
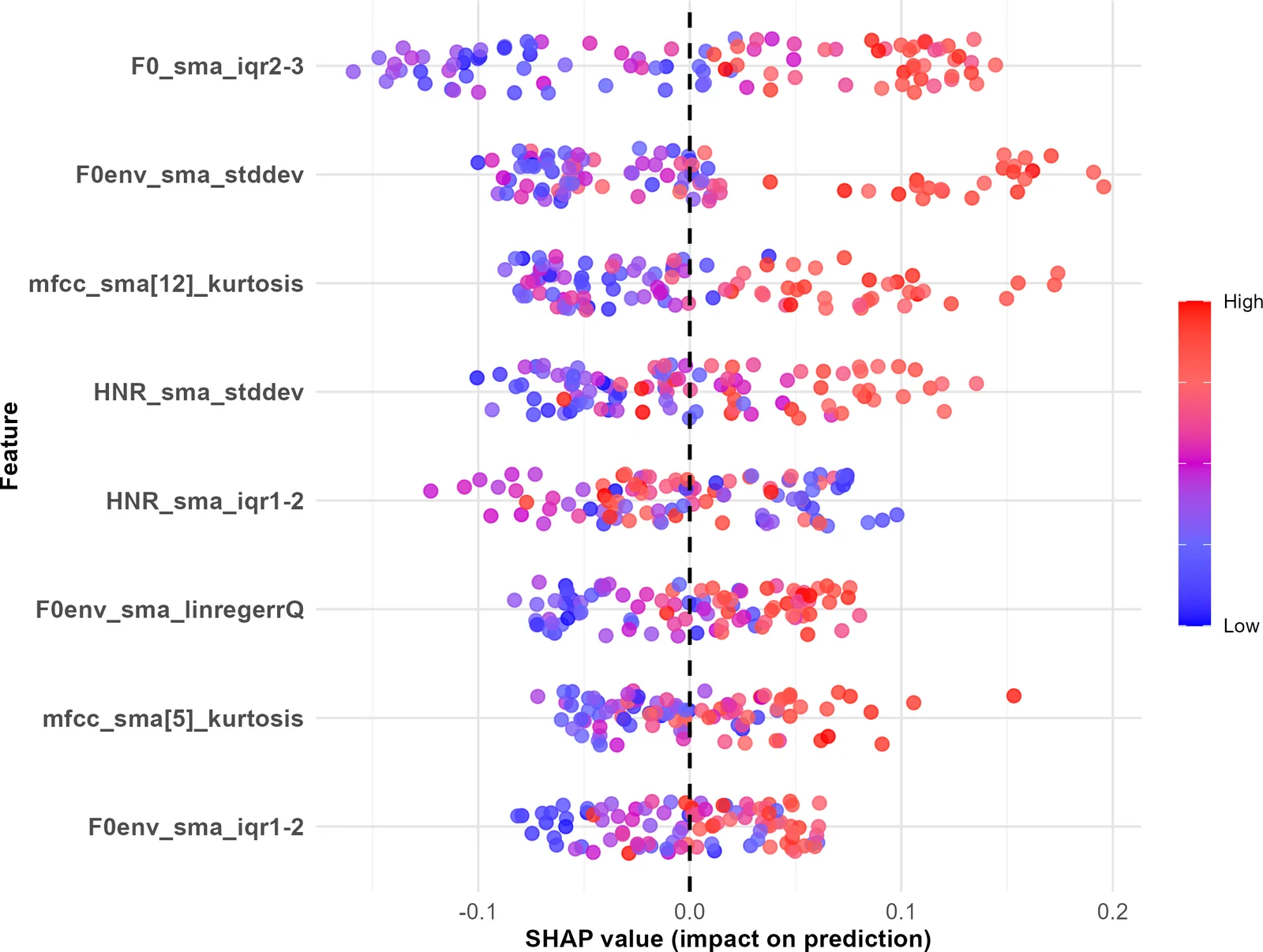
Shapley additive explanations (SHAP) summary plot depicting the effects and directionality of features.

Shapley additive explanations analyses provided convergent evidence regarding the prominence of the robust feature set (Figure S5 in Multimedia Appendix 1; Figure 3). F0_sma_iqr2-3 and F0env_sma_stddev generally contributed positively to SI predictions, whereas HNR-related features tended to contribute negatively. Notably, mfcc_sma [12]_kurtosis showed bidirectional Shapley additive explanations values (−0.087 to 0.174), and F0env_sma_stddev exhibited the largest spread (−0.100 to 0.196), indicating heterogeneity in feature contributions across individuals.

## Discussion

### Principal Findings

This study presents a methodologically rigorous proof-of-concept framework for detecting SI from standardized, smartphone-recorded read speech in university students. Speech offers a low-burden complementary behavioral signal that may mitigate reliance on self-disclosure in SI assessment [35]. The central contribution is a leakage-resistant modeling and validation pipeline built around (1) standardized prompts, (2) explicit reading compliance checks, (3) participant-level cross-validation to prevent within-speaker contamination, and (4) transparent feature stability and interpretability analyses. These design choices directly address key validity threats in speech-based modeling, particularly speaker leakage and evaluation bias.

While our results are promising, the interpretation must be matched to our specific methodology and sample characteristics. Notably, the SI group was significantly older than the control group (*P*=.001). Although this age difference was numerically small (approximately 1 year) and occurred within a narrow developmental window (late adolescence), it represents a potential confounding factor that limits the direct attribution of all acoustic variance to psychological status alone. Furthermore, our use of a constrained read speech task, while it improved standardization, may have limited the expression of more intense, spontaneous emotional signals often captured in crisis hotline studies.

The best-performing model in terms of AUC was RF (AUC=0.813; accuracy=0.748), followed by NB (AUC=0.806; accuracy=0.757). The observed misclassification rate—particularly the false negatives—merits clinical consideration. Misclassification in SI detection may stem from several factors: (1) the “silent” or “stoic” phenotype of ideation where vocal prosody remains intact despite internal distress; (2) individual variability in baseline speaking styles that overlaps with SI-linked features; and (3) the modest sample size (N=96), which may not capture the full heterogeneity of vocal expressions in SI. Consequently, our model should be viewed as a screening aid rather than a definitive diagnostic tool, consistent with the need for cautious deployment of AI in mental health.

These results fall within the performance range reported in several influential speech-suicide machine learning studies, although differences in population, speech task, labels, and validation design constrain direct numeric comparisons. Su et al [22] analyzed crisis hotline calls (naturalistic, emotionally loaded speech with clinician-rated risk labels) and reported the best model performance in terms of accuracy at approximately 0.75, with *F*_1_-scores of approximately 0.70. Belouali et al [36] used mobile app narrative recordings in US veterans and reported RF performance of approximately 0.80 (AUC) with sensitivity of 86% and specificity of 70% when combining acoustic and linguistic features. Min et al [21] evaluated voice from clinical interviews in patients with mood disorders and observed more modest between-person discrimination (AUC≈0.62) but improved within-person prediction of worsening suicidality over time (accuracy≈0.79; AUC≈0.67), highlighting how label definition (cross-sectional discrimination vs longitudinal change) and recording context can shift achievable performance. Pillai et al [37] further demonstrated that cross-dataset generalization is frequently poor even when within-dataset metrics appear strong, indicating that distribution shift must be considered alongside algorithm choice.

Compared with the standardized reading tasks widely used in suicide prediction research based on acoustic features, such as the rainbow passage [38,39], our standardized reading materials incorporated a wider variety of sentence structures and sentence functions. The sentences were relatively more independent from one another and exhibited greater variability across items. Furthermore, compared with the reading passages commonly used in English-speaking contexts, our materials were better suited to Chinese phonological characteristics, thereby providing a standardized reading task for populations with Chinese linguistic backgrounds.

In this context, the observation that a brief read speech task—more constrained than narratives or crisis calls—still yielded discrimination comparable to that of several naturalistic speech approaches suggests that standardization can retain clinically relevant acoustic variability while improving controllability and reproducibility [15].

A major methodological strength of the present work is the explicit handling of within-participant leakage introduced by multiple recordings per speaker (16 utterances per participant). Leakage is a known failure mode in speech machine learning: if folds are split by recordings rather than participants, models can inadvertently learn speaker identity or stable vocal traits, inflating apparent performance [40]. Participant-level stratified cross-validation—where all utterances from a participant remain in one fold—directly addressed this issue and is consistent with best practice recommendations in behavioral machine learning and speech-based mental health research. In addition, the compliance pipeline (automatic transcription plus fuzzy alignment and manual review) reduced noise from off-prompt or incomplete readings, which can otherwise obscure acoustic signal in smartphone audio collected outside laboratory conditions. Such quality control procedures are often underdescribed in the literature but are likely to become increasingly important as speech data collection shifts toward real-world mobile settings [36].

The feature patterns most consistently associated with SI—indexes of pitch variability (F0-related measures), spectral shape (MFCC kurtosis), and harmonicity (HNR)—are broadly consistent with prior work linking suicidality and related affective states to altered prosody and voice quality while also illustrating how different speech tasks may privilege different acoustic cues. In hotline settings, spontaneous emotional speech likely amplifies prosodic instability and arousal-linked voice changes, whereas standardized reading reduces linguistic variability and may highlight subtler motor or phonatory correlates [22]. In narrative recordings, acoustic and linguistic features jointly contribute to psychiatric classification, suggesting that, when free speech is available, content-related information can be additive; standardized prompts isolate acoustics, making inferred associations more interpretable as vocal correlates rather than mixed vocal-semantic signals [25,41,42]. Reviews of speech processing in depression and suicidality similarly emphasize recurrent involvement of pitch, energy, spectral measures (often MFCC derived), and voice quality features while noting that effect direction and magnitude can vary by task, language, and recording conditions [15].

The comparability of performance estimates across studies depends not only on metrics but also on sampling strategies and outcome construction. Hotline and telehealth studies often involve higher base rates of distress and labels reflecting clinician-rated risk strata rather than strictly defined ideation thresholds, which can influence separability and reported metrics [22]. Conversely, balanced case-control designs improve statistical efficiency for early-stage method development but depart from community prevalence distributions. Consequently, accuracy in a balanced sample should not be interpreted as an expected positive predictive value for population screening without recalibration [43]. These considerations underscore the importance of transparent reporting and leakage-aware validation, and they help explain why methodological heterogeneity in recording contexts and analytic pipelines continues to challenge transportability across settings [20].

Methodologically, the pattern observed in this study—strong performance of simpler, well-regularized models (RF and NB) alongside weaker performance of the neural network—aligns with prior observations that more complex architectures can overfit in small, heterogeneous datasets without extensive harmonization [21]. Consistent with this, cross-dataset evidence indicates that robustness under distribution shift remains a central bottleneck even when within-dataset metrics appear strong, highlighting the need for evaluation designs and dataset diversity that explicitly test generalizability [37].

In summary, the present work contributes a reproducible methodological template for SI detection from voice by combining standardized prompts, compliance quality control, leakage-aware participant-level validation, and transparent feature stability analyses. When viewed alongside hotline, clinical interview, and narrative speech studies, these results support the feasibility of extracting suicidality-relevant information from speech across diverse contexts while underscoring the importance of rigorous evaluation and cross-context testing as the next step for the field.

### Limitations

Several limitations merit consideration. First, the cross-sectional nature and specific recruitment from a single university limit the generalizability of our findings. The identified acoustic markers should be interpreted as correlational signals rather than markers of future suicide risk. As highlighted by the issue of misclassification in our sample, these features alone are insufficient for high-stakes clinical decision-making without integration into a broader, multimodal assessment framework. Second, while the standardized read speech task controlled linguistic content and reduced between-speaker variability, it may have constrained the range of emotional and expressive speech characteristics captured. Accordingly, the ecological validity of the identified acoustic patterns may be lower than that achievable with spontaneous or emotion-elicited speech. Third, the neural network–based model showed weaker performance than the simpler models in this dataset, plausibly reflecting the moderate sample size and the challenges that complex models face in learning stable, generalizable representations from heterogeneous speech data. Fourth, our pipeline relies exclusively on acoustic features derived from standardized read speech, ignoring linguistic content and physiological biomarkers. These limitations are consistent with concerns raised in recent reviews and empirical work on acoustic-based suicide risk assessment [20].

### Future Directions

Future research should strengthen the construct validity of acoustic indicators and evaluate their sensitivity to individual differences in SI. First, expanding recruitment to multiple universities and larger samples will improve statistical power and enable testing of whether the identified acoustic features consistently reflect underlying psychological constructs—such as emotional dysregulation, cognitive constriction, or motivational withdrawal—across diverse student populations. Second, future studies should explicitly model individual differences in speech production and psychological functioning. Beyond group-level classification, person-centered and longitudinal designs may help disentangle stable, trait-like vocal characteristics from state-dependent fluctuations associated with changes in suicidal risk, thereby improving interpretability and reducing misclassification driven by normative speaking style variability. Third, efforts to develop early warning systems should remain grounded in psychological theory and measurement principles. Acoustic features are best evaluated as indicators within a multi-method assessment framework, complementing self-report and clinical judgment rather than serving as stand-alone predictors. Aligning model outputs with interpretable psychological constructs may facilitate responsible integration into prevention contexts while preserving conceptual clarity and ethical safeguards. Fourth, moving beyond the unimodal acoustic approach, future research should explore multimodal fusion that integrates speech acoustics with natural language processing and physiological biomarkers. Multimodal fusion may be a superior method for detecting SI as it captures complementary risk dimensions—such as lexical markers of hopelessness, prosodic instability, and autonomic arousal—while compensating for the limitations of any single modality.

### Conclusions

This study demonstrates the feasibility of using smartphone-acquired acoustic features from standardized read speech to discriminate SI among Chinese university students. The RF model showed promising preliminary performance (AUC=0.813) and identified stable acoustic markers. These findings support speech-derived acoustics as a low-burden, objective complement to traditional self-report screening in campus mental health contexts, where concealment and resource constraints can limit detection. Importantly, acoustic indicators should be interpreted as correlational signals rather than diagnostic tools, and their use should be embedded within existing counseling pathways and ethical safeguards. With further external validation and evaluation in more diverse, real-world settings, speech-based acoustic analysis can potentially alleviate the burden on campus counseling centers by providing an automated, preliminary triage stage in a multimodal screening framework.

## Supplementary material

10.2196/92646 Multimedia Appendix 1 Supplementary materials including 5 figures, 4 tables, and 1 Excel file (list of acoustic feature names).

## Abbreviations

AUC: area under the curve
F0: fundamental frequency
FDR: false discovery rate
GLMNET: elastic net generalized linear model
HNR: harmonic-to-noise ratio
KNN: k-nearest neighbor
LSP: Line Spectrum Pairs
MFCC: mel-frequency cepstral coefficient
NB: naive Bayes
NNET: feed-forward neural network
OR: odds ratio
RF: random forest
SI: suicidal ideation
SIOSS: Self-Rating Idea of Suicide Scale
SVM: support vector machine
XGB: Extreme Gradient Boosting

## References

[1] (2025). Suicide. World Health Organization.

[2] Lamontagne SJ, Zabala PK, Zarate CA Jr, Ballard ED (2023). Toward objective characterizations of suicide risk: a narrative review of laboratory-based cognitive and behavioral tasks. Neurosci Biobehav Rev.

[3] Sommers-Flanagan J, Shaw SL (2017). Suicide risk assessment: what psychologists should know. Prof Psychol Res Pract.

[4] Hamdan-Mansour AM, Hamdan-Mansour RA, Allaham DM, Alrashidi M, Alhaiti A, Mansour LA (2024). Academic procrastination, loneliness, and academic anxiety as predictors of suicidality among university students. Int J Ment Health Nurs.

[5] Akram U, Ypsilanti A, Gardani M (2020). Prevalence and psychiatric correlates of suicidal ideation in UK university students. J Affect Disord.

[6] Akram U, Irvine K, Gardani M, Allen S, Akram A, Stevenson JC (2023). Prevalence of anxiety, depression, mania, insomnia, stress, suicidal ideation, psychotic experiences, & loneliness in UK university students. Sci Data.

[7] Akram U, Drabble J, Irvine K (2025). Prevalence and psychiatric correlates of loneliness in UK university students. Npj Ment Health Res.

[8] Wang J, Yang Y, Chen Y (2025). Loneliness, internalizing and externalizing problems, and suicidal ideation among Chinese adolescents: a longitudinal mediation analysis. J Adolesc Health.

[9] Yang T, He Y, Wu L (2023). The relationships between anxiety and suicidal ideation and between depression and suicidal ideation among Chinese college students: a network analysis. Heliyon.

[10] Bornheimer LA, Czyz E, Koo HJ (2022). Suicide risk profiles and barriers to professional help-seeking among college students with elevated risk for suicide. J Psychiatr Res.

[11] Chu H, Yang Y, Zhou J (2021). Social support and suicide risk among Chinese university students: a mental health perspective. Front Public Health.

[12] Zhao M, Xin Y, Bai X (2025). Risk factors for suicidality among college students: a systematic review and meta-analysis. J Affect Disord.

[13] Deng L, Lee C, Lee S, Ding Y, Newman G (2025). Suicide risks among U.S. college students: a time-series cross-sectional study examining institutional characteristics and behavioral factors. Prev Sci.

[14] Lázaro-Pérez C, Munuera Gómez P, Martínez-López JÁ, Gómez-Galán J (2023). Predictive factors of suicidal ideation in Spanish university students: a health, preventive, social, and cultural approach. J Clin Med.

[15] Cummins N, Scherer S, Krajewski J, Schnieder S, Epps J, Quatieri TF (2015). A review of depression and suicide risk assessment using speech analysis. Speech Commun.

[16] Figueroa C, Guillén V, Huenupán F (2024). Comparison of acoustic parameters of voice and speech according to vowel type and suicidal risk in adolescents. J Voice.

[17] Kappen M, Vanhollebeke G, Van Der Donckt J, Van Hoecke S, Vanderhasselt MA (2024). Acoustic and prosodic speech features reflect physiological stress but not isolated negative affect: a multi-paradigm study on psychosocial stressors. Sci Rep.

[18] Lin Q, Zhang J, Wang W, Tan C, Wu X, Zhao J (2025). A machine learning-based case-control study on suicide risk identification: integrating acoustic and linguistic features under stress conditions. Depress Anxiety.

[19] MacPherson MK, Abur D, Stepp CE (2017). Acoustic measures of voice and physiologic measures of autonomic arousal during speech as a function of cognitive load. J Voice.

[20] Marie A, Garnier M, Bertin T (2026). Acoustic and machine learning methods for speech-based suicide risk assessment: a systematic review. J Affect Disord.

[21] Min S, Shin D, Rhee SJ (2023). Acoustic analysis of speech for screening for suicide risk: machine learning classifiers for between- and within-person evaluation of suicidality. J Med Internet Res.

[22] Su Z, Jiang H, Yang Y, Hou X, Su Y, Yang L (2025). Acoustic features for identifying suicide risk in crisis hotline callers: machine learning approach. J Med Internet Res.

[23] Krautz AE, Volkening J, Raue J (2025). Prediction of suicide using web based voice recordings analyzed by artificial intelligence. Sci Rep.

[24] Ding Z, Zhou Y, Dai AJ (2025). Speech based suicide risk recognition for crisis intervention hotlines using explainable multi-task learning. J Affect Disord.

[25] Zhu Y, Yin Q, Xu H (2025). Speech feature identification model for depressed individuals with suicidal ideation based on autobiographical memory. BMC Psychiatry.

[26] Stasak B, Epps J, Schatten HT, Miller IW, Provost EM, Armey MF (2021). Read speech voice quality and disfluency in individuals with recent suicidal ideation or suicide attempt. Speech Commun.

[27] Torous J, Andersson G, Bertagnoli A (2019). Towards a consensus around standards for smartphone apps and digital mental health. World Psychiatry.

[28] (2025). Ethics and governance of artificial intelligence for health: guidance on large multi-modal models. World Health Organization.

[29] Eyben F, Wöllmer M, Schuller B (2010). MM ’10: Proceedings of the 18th ACM International Conference on Multimedia.

[30] Titze IR (1989). Physiologic and acoustic differences between male and female voices. J Acoust Soc Am.

[31] Yücesoy E (2024). Gender recognition based on the stacking of different acoustic features. Appl Sci.

[32] Albaldawi WS, Almuttairi RM (2021). Hybrid ANOVA and LASSO methods for feature selection and linear support vector, multilayer perceptron and random forest classifiers based on Spark environment for microarray data classification. IOP Conf Ser Mater Sci Eng.

[33] Yu Z, Zhang X (2017). A high-value mobile communication user prediction method based on effective feature selection. J Wuhan Univ Sci Technol.

[34] Nogueira S, Sechidis K, Brown G (2018). On the stability of feature selection algorithms. J Mach Learn Res.

[35] Obegi JH (2021). How common is recent denial of suicidal ideation among ideators, attempters, and suicide decedents? A literature review. Gen Hosp Psychiatry.

[36] Belouali A, Gupta S, Sourirajan V (2021). Acoustic and language analysis of speech for suicidal ideation among US veterans. BioData Min.

[37] Pillai A, Nepal SK, Wang W (2023). Investigating generalizability of speech-based suicidal ideation detection using mobile phones. Proc ACM Interact Mob Wearable Ubiquitous Technol.

[38] Wahidah NN, Wilkes MD, Salomon RM (2015). Timing patterns of speech as potential indicators of near-term suicidal risk. Int J Multidiscip Curr Res.

[39] Yingthawornsuk T, Shiavi RG (2008). 2008 International Conference on Control, Automation and Systems.

[40] Low DM, Bentley KH, Ghosh SS (2020). Automated assessment of psychiatric disorders using speech: a systematic review. Laryngoscope Investig Otolaryngol.

[41] Dobbs MF, McGowan A, Selloni A (2023). Linguistic correlates of suicidal ideation in youth at clinical high-risk for psychosis. Schizophr Res.

[42] Crocamo C, Cioni RM, Canestro A (2025). Acoustic and natural language markers for bipolar disorder: a pilot, mHealth cross-sectional study. JMIR Form Res.

[43] Posner K, Brown GK, Stanley B (2011). The Columbia-Suicide Severity Rating Scale: initial validity and internal consistency findings from three multisite studies with adolescents and adults. Am J Psychiatry.

