# Supplementary material for: A Smartphone-Based Acoustic Machine Learning Pipeline for Detecting Suicidal Ideation: Case-Control Model Development and Validation Study
Source: JMIR Form Res. 2026 Sep 15;10:e92646. doi: 10.2196/92646 (PMC13577659; doi:10.2196/92646)
Supplement: Supplementary file 1 [file formative-v10-e92646-s001.docx]

**Supplementary Materials**

**Supplementary Figures**

*Figure S1. Frequency of feature selection across cross-validation folds.*

*Figure S2. Mixed-effects model results for acoustic features.*

*Figure S3. Feature importance from the Random Forest model.*

*Figure S4. Partial dependence plots (PDPs) of key acoustic features.*

*Figure S5. Feature importance from Random Forest model.*

**Supplementary Tables**

Table S1. Overview of the Final Sentence

| **No.** | **Sentence** | **Sentence Mood** | **Sentence Function** | **Pleasure** | **Arousal** | **Dominance** | **Source** |
| --- | --- | --- | --- | --- | --- | --- | --- |
| 1 | “Do not impose on others what you yourself do not desire,” and “enter and depart as friends, watching over and assisting one another” — these reflect the “harmony” aspect of the core socialist values. | Declarative Sentence | Cognitive Function | 7.24 | 4.54 | 7.15 | Expert Evaluation |
| 2 | “The rise and fall of the nation is every citizen’s responsibility,” “a gentleman is broad‑minded while a petty person is anxious,” “one’s words must be kept and actions resolute,” and “the benevolent love others, the courteous respect others” — these proverbs exemplify the core socialist values of patriotism, dedication, integrity, and friendliness. |  |  | 7.37 | 4.74 | 7.02 |  |
| 3 | I despise my own family; it is one that saps one’s will to live(Shi, 2022). |  |  | 2.89 | 5.83 | 5.15 | Literature Source |
| 4 | I stood at a shabby podium and delivered a constrained speech with a face full of sorrow(Shi, 2022). |  |  | 2.43 | 6.15 | 4.35 |  |
| 5 | The grass is green.(Stasak et al., 2021) |  |  | 7.39 | 3.22 | 7.20 |  |
| 6 | On the glacier, the azure lake lay as calm and reflective as a mirror. |  |  | 7.59 | 3.33 | 7.26 | Expert Evaluation |
| 7 | Our country is making great strides toward prosperity, democracy, civility, and harmony—surely that is something to be proud of? | Rhetorical Question | Emotional Function | 7.63 | 5.70 | 6.83 |  |
| 8 | Why am I always so timid and helpless? |  |  | 2.81 | 6.44 | 4.26 |  |
| 9 | Hasn’t he always handled issues with steadfast composure? |  |  | 4.93 | 4.65 | 5.98 |  |
| 10 | What an exhilarating society of freedom, equality, justice, and rule of law! | Exclamatory Sentence |  | 7.30 | 5.19 | 6.76 |  |
| 11 | Everything feels so humiliating! |  |  | 2.70 | 6.52 | 4.78 |  |
| 12 | Tonight’s moonlight and breeze truly calm the spirit! |  |  | 7.85 | 3.81 | 7.52 |  |
| 13 | I truly wish that I were already dead.(Stasak et al., 2021) | Declarative Sentence | Suicide-related Expression |  | | | Literature Source |
| 14 | The world would be better off without me(Macdonald et al., 2024). |  |  |  |  |  |  |
| 15 | I felt an overwhelming sense of despair—what light remains in my life?(Macdonald et al., 2024) | Rhetorical Question |  |  |  |  |  |
| 16 | I feel like a giant sponge that has absorbed all the joy in the world, leaving only pain!(Macdonald et al., 2024) | Exclamatory Sentence |  |  |  |  |  |

Table S2. Group Differences in Acoustic Features Between Suicide and Control Groups

| **Feature** | **Control(M)** | **Suicide(M)** | **T** | **P** | **Cohen_d** | **P-FDR** |
| --- | --- | --- | --- | --- | --- | --- |
| F0_sma_iqr2-3 | 74.217 | 103.161 | -4.168 | 0.000 | 0.851 | 0.001 |
| F0env_sma_stddev | 83.450 | 94.165 | -3.922 | 0.000 | 0.801 | 0.001 |
| F0env_sma_iqr1-2 | 44.514 | 58.531 | -3.311 | 0.001 | 0.676 | 0.004 |
| F0env_sma_linregerrQ | 5562.383 | 6609.062 | -3.209 | 0.002 | 0.655 | 0.004 |
| mfcc_sma[5]_kurtosis | 2.985 | 3.193 | -3.008 | 0.003 | 0.614 | 0.005 |
| mfcc_sma[12]_kurtosis | 3.045 | 3.257 | -2.778 | 0.007 | 0.567 | 0.009 |
| HNR_sma_iqr1-2 | 11.690 | 10.124 | 2.160 | 0.033 | -0.441 | 0.038 |
| HNR_sma_stddev | 64.812 | 73.505 | -1.610 | 0.111 | 0.329 | 0.111 |

Table S3. Correlations Between Acoustic Features and Suicide Status

| **Feature** | **Correlation coefficient** | **P** | **P-FDR** |
| --- | --- | --- | --- |
| F0_sma_iqr2-3 | 0.395 | 0.000 | 0.001 |
| F0env_sma_stddev | 0.375 | 0.000 | 0.001 |
| F0env_sma_iqr1-2 | 0.323 | 0.001 | 0.004 |
| F0env_sma_linregerrQ | 0.314 | 0.002 | 0.004 |
| mfcc_sma[5]_kurtosis | 0.296 | 0.003 | 0.005 |
| mfcc_sma[12]_kurtosis | 0.276 | 0.007 | 0.009 |
| HNR_sma_iqr1-2 | -0.217 | 0.033 | 0.038 |
| HNR_sma_stddev | 0.164 | 0.111 | 0.111 |

Table S4. Logistic Regression Results Predicting Suicide Status From Acoustic Features

| **Feature** | **Data_Type** | **Beta** | **Odds_Ratio** | **CI_Lower** | **CI_Upper** | **P** | **P_FDR** |
| --- | --- | --- | --- | --- | --- | --- | --- |
| F0_sma_iqr2-3 | raw | 0.025 | 1.025 | 1.011 | 1.039 | 0.000 | 0.002 |
| F0env_sma_stddev | raw | 0.059 | 1.061 | 1.026 | 1.097 | 0.001 | 0.002 |
| F0env_sma_iqr1-2 | raw | 0.033 | 1.033 | 1.012 | 1.056 | 0.002 | 0.007 |
| F0env_sma_linregerrQ | raw | 0.000 | 1.000 | 1.000 | 1.001 | 0.003 | 0.007 |
| mfcc_sma[5]_kurtosis | raw | 1.869 | 6.485 | 1.725 | 24.383 | 0.006 | 0.009 |
| mfcc_sma[12]_kurtosis | raw | 2.088 | 8.067 | 1.721 | 37.811 | 0.008 | 0.011 |
| HNR_sma_iqr1-2 | raw | -0.133 | 0.876 | 0.772 | 0.994 | 0.040 | 0.046 |
| HNR_sma_stddev | raw | 0.013 | 1.013 | 0.997 | 1.029 | 0.112 | 0.112 |
| F0_sma_iqr2-3 | standardization | 0.917 | 2.502 | 1.523 | 4.113 | 0.000 | 0.002 |
| F0env_sma_stddev | standardization | 0.853 | 2.346 | 1.448 | 3.801 | 0.001 | 0.002 |
| F0env_sma_iqr1-2 | standardization | 0.716 | 2.046 | 1.286 | 3.255 | 0.002 | 0.007 |
| F0env_sma_linregerrQ | standardization | 0.710 | 2.034 | 1.265 | 3.272 | 0.003 | 0.007 |
| mfcc_sma[5]_kurtosis | standardization | 0.662 | 1.938 | 1.213 | 3.097 | 0.006 | 0.009 |
| mfcc_sma[12]_kurtosis | standardization | 0.811 | 2.249 | 1.235 | 4.097 | 0.008 | 0.011 |
| HNR_sma_iqr1-2 | standardization | -0.480 | 0.619 | 0.392 | 0.978 | 0.040 | 0.046 |
| HNR_sma_stddev | standardization | 0.337 | 1.400 | 0.924 | 2.122 | 0.112 | 0.112 |

Supplementary Data S1
